# Parkinson's disease constipation effect of electroacupuncture at ST25 through colonic motility and enteric neuropathology

**DOI:** 10.3389/fneur.2022.1092127

**Published:** 2023-01-11

**Authors:** Li-zhe-xiong Song, Yuan Li, Xu Qian, Na Xu, Zhi Yu, Jing-wen Dai, Bin Xu, Xuan-ming Hu

**Affiliations:** ^1^Key Laboratory of Acupuncture and Medicine Research of Ministry of Education, Nanjing University of Chinese Medicine, Nanjing, China; ^2^School of Acupuncture-Moxibustion and Tuina, Nanjing University of Chinese Medicine, Nanjing, China; ^3^Acupuncture Department, Nanjing Hospital of Traditional Chinese Medicine, Affiliated to Nanjing University of Traditional Chinese Medicine, Nanjing, China

**Keywords:** Parkinson's disease constipation, electroacupuncture, enteric nervous system, neuropathology, colonic motility

## Abstract

**Background:**

The enteric nervous system (ENS) plays a central role in developing Parkinson's disease (PD) constipation, and the regulation of the ENS may be a key component in treating PD constipation. Electroacupuncture (EA) can effectively treat constipation symptoms in PD, but research on its specific mechanisms, especially in terms of ENS, is relatively lacking. Therefore, we investigated whether EA at ST25 promotes the restoration of ENS structure and colonic motor function in the rotenone-induced PD constipation rat model.

**Methods:**

In this study, we evaluated constipation symptoms by stool characteristics, excretion and water volume, and whole gut transit time and observed colonic motility regulation through colonic motion detection and pathological changes in the colonic myenteric nervous plexus by transmission electron microscopy and immunofluorescence staining.

**Results:**

EA significantly improved the constipation symptoms and positively adjusted the colonic motility in rotenone-induced PD constipation rats. At the same time, EA reversed the rotenone-induced colonic myenteric nervous plexus injury and regulated the ratio of inhibitory and excitatory neurotransmitters.

**Conclusion:**

Our results indicate that EA treatment of PD constipation may be mediated through the adjustment of ENS.

## Introduction

Parkinson's disease (PD) is a common neurodegenerative disorder characterized by motor and non-motor symptoms (NMS), both of which are associated with increasing age and the course of the disease. Gastrointestinal dysfunction symptoms are one of the most common forms of NMS in PD (1). Among all gastrointestinal symptoms, constipation is the most common manifestation of lower gastrointestinal dysfunction in patients with PD, with a prevalence of 24.6–63% (2). Recent evidence indicates that constipation may also be one of the most common disorders in the prodromal phase of PD, highlighting its value as a risk factor or predictor of the development of PD (3). The currently available evidence suggests the presence of α-synaptic nuclear protein (α-syn) aggregates and neurotransmitter alterations in intestinal tissue. All these findings support Braak's proposed pathophysiological model of α-syn aggregates in Parkinson's disease, which is the early pathological involvement of the enteric nervous system (ENS) and the dorsal motor nucleus vagus (4, 5). The ENS plays a crucial role in the neurodegenerative process leading to PD (6). The ENS determines the motor patterns of the gastrointestinal tract, processes sensory input from mechanical and chemical receptors in the gut wall, and integrates sympathetic and parasympathetic inputs, interacting with the immune and endocrine systems of the gut to produce coordinated effects (7). All these functions are based on the interaction between the different neuronal subtypes in the ENS, and the balance between the type of input and the level of postsynaptic receptor expression released into the neuronal network by each neurotransmitter (8).

While much progress has been made in understanding the pathogenesis of PD and the symptomatic treatment of PD-related symptoms, there are currently no effective neuroprotective or disease-modifying therapies to slow the progression of the disease. The physical, psychological, social, and economic burden of PD remains the most challenging barrier to treatment, especially in the advanced stages of the disease (9). Treatments for constipation in patients with PD include behavioral changes (e.g., increased water intake and physical activity) and the use of pro-secretory agents or osmotic laxatives. In addition, complementary and alternative therapies combined with TCM such as acupuncture, Tui Na, and Tai Chi are increasingly used in PD (10, 11). In recent years, acupuncture has received increasing attention as a non-invasive treatment method. A growing number of studies have investigated the effectiveness of acupuncture targeting PD and other related disorders, such as motor dysfunction (12–14), anxiety (15), depression (16), insomnia (17), and constipation (18, 19), with some positive results. Although existing systematic evaluations and meta-analyses have shown conflicting results for acupuncture for PD constipation due to significant heterogeneity and small sample sizes (20–24), the fact that acupuncture was considered an effective or safe treatment for functional constipation and gastrointestinal disorders (25, 26) in some randomized trials. Available evidence suggests that acupuncture treatment has the potential to alleviate motor and NMS of PD, but the underlying mechanisms are unclear (27–29). The neuroprotective effects of acupuncture on neurodegenerative lesions in animal models of PD are mainly focused on cerebral neurons (30–32), and no studies have yet reported the effects of acupuncture on the ENS. Here, we investigate the mechanisms of acupuncture to alleviate bowel dysfunction in PD constipation by revealing the effects of acupuncture treatment on the ENS myenteric nervous plexus and its neurotransmitters in an animal model of PD.

## Materials and methods

### Establishment of the experimental animal model

In this study, 8-week-old Sprague Dawley (SD) rats were supplied by the Beijing Vital River Laboratory Animal Technology Co., Ltd. [No. 110011220101889264, under grant SCXK(JING)2021-0011]. The experimental rats were kept in a barrier environment with stable parameters (conditions: 12/12-h light/dark cycle; temperature, 22 ± 2°C; relative humidity 60 ± 5%). The animals were randomly numbered and divided into three groups: model group, electroacupuncture (EA) group, and control group (for convenience, the following texts refer to them as PD, EA, and SH groups, respectively), with six animals in each group. They were kept in cages of the same size in groups and had free access to food and water. PD was induced by giving a low dose of rotenone. The PD and EA groups were injected subcutaneously with rotenone solvent on the back of the neck at a dose of 0.1 ml/kg once a day for 5 days a week. The solvent was prepared by dissolving 200 mg of rotenone (M6209; Abmole Bioscience Inc, Houston, TX, USA) in 3 ml of dimethyl sulfoxide (DMSO, D8370; Beijing Solarbio Science & Technology, Tongzhou, Beijing, China) and then fixed to 100 ml with sunflower oil to make up 2 mg/ml of rotenone sunflower oil solvent. The SH group was injected with an equal volume of solvent mixture (3% DMSO sunflower oil solvent).

**Figure 1:**
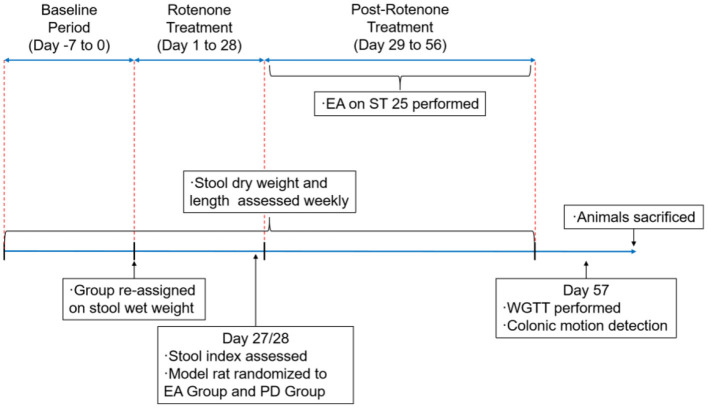
Schedule of the experimental procedures. EA, electroacupuncture; PD group, the model group; ST25, acupoint Tianshu; WGTT, whole gut transit time.

Weekly metabolic cages were performed after rotenone injection to measure dry weight and length of stool (the specific methods of metabolic cage method and stool collection and analysis will be elaborated in Stool Collection and Analysis). The modeled rats were evaluated after 4 weeks of rotenone treatment. Rats with defecation indexes (stool dry weight and length) lower than the SH group mean and PD behavioral score of ≥2 were considered to meet the criteria. The process of the present study is shown in Figure 1. All the experiments were approved by the Scientific Investigation Board of the Nanjing University of Traditional Chinese Medicine, Nanjing , China (permission no. 202112A047) and performed per the Principles of Laboratory Animal Care and the Guide for the Care and Use of Laboratory Animals published by the National Science Council, China (under grant 202006A016).

### PD behavioral evaluation

Parkinson's disease behavioral evaluation was performed according to the criteria developed by Chen et al. (33). The specific scoring criteria are shown in Table 1. Only rats with a behavioral score of ≥2 were included in the next step of screening for defecation indicators because rats with a behavioral score of ≥2 have relatively significant neurological deficits and the PD rat models in this range are more reliable (34).

**Table 1:** PD behavioral evaluation criteria. The higher score includes the lower scoring performance.

**Score**	**Performance**
1	Reduced refusal behavior, yellowing and soiling of the coat, arching of the back, erect hair, and reduced active activity
2	Significantly reduced active movement, slow movement, tremor, or unstable gait
4	Unsteady gait, or the inability to walk in a straight line, or swiveling to one side when walking
6	Reclining to one side, unilateral forelimb and/or hindlimb paralysis, difficulty in walking, difficulty in eating
8	Complete unilateral paralysis of the forelimb and/or hind limb, contracture of the limbs, significant weight loss, inability to eat
10	Dying state or death

### Stool collection and analysis

Rat stools were collected using the metabolic cage method as follows. The rats were placed in individual wire cages with a separation device, which consisted of two layers of separation nets, the upper layer of which separated the rats from the stools to ensure that the stools and the rats' activities will not interact, and the lower layer of which separated the stools from the urine to ensure that the wet weight of the stools will not be affected by the urine. Rats' stools were collected at the end of the first 5 days of treatment each week, and the time was fixed from 7:00 p.m. to 7:00 a.m. for a 12-h period to reduce the error caused by water evaporation.

The stools were collected in sealed bags in the same order as the rats were placed. Then, the stools were dried in a dryer (70°C, 6 h; Septree ST-06; Xinchi e-commerce, Foshan, Guangdong, China) until the weight no longer changed, and the dry weight of the stools was weighed using an analytical balance (QUINTIX313-1CN; Sartorius Scientific Instruments, Shunyi, Beijing, China). The dry–wet difference (ΔW) of the stools is expressed as Wet weight (g)–Dry weight (g). Finally, the stools were arranged by long diameter on a grid paper with a scale to calculate their length.

### Whole gut transit time

Whole gut transit time (WGTT) was determined by the interval between the oral gavage of 3 ml of 0.5% phenol red (dissolved in PBS) and the first appearance in stools of red marker (35, 36). The rats after the oral gavage of phenol red were observed visually for the stools and the color of the stools. The actual presence of phenol red in the fecal sample was confirmed by spectrophotometric analysis. As described previously (37), we learned that the WGTT lasted more than 8 h; therefore, starting from the seventh hour after the oral phenol red, the animal cages were checked every 10 min for red in the stools.

### Colonic motion detection

**Figure 2:**
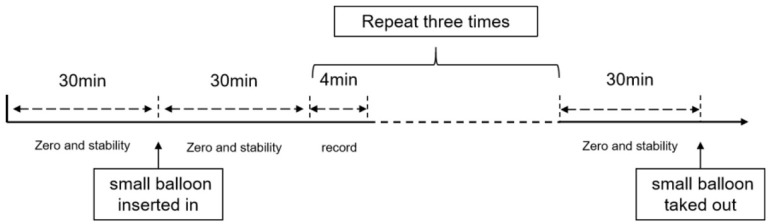
Schedule of colonic motion detection.

The animals were fasted overnight with free access to water and gas anesthesia with isoflurane (2–5%; 9020000522; Shenzhen Ruiwode Lift Technology, Nanshan, Shenzhen, China), and colonic motility was recorded using a previously described method (38). A small balloon made of flexible condom rubber was inserted into the colon 3–6 cm *via* the anus of rats. The pressure in the balloon was measured with a transducer and recorded with a physiological signal-acquisition system (AD Instruments, Pudong, Shanghai, China) for further analysis. Zeroing was performed before and after balloon placement into the animal for 30 min, and *in vitro* zeroing was performed in water. After baseline stabilization, the recording was started. After recording, the balloons were removed and placed in water for re-zeroing to compare with the pre-zeroing data and to prepare for the next animal. After all the animals were finished, the data were processed by LabChart8 software, and the data were randomly selected for three discrete periods of 4 min each. Frequency (Hz), mean (Kpa), min (Kpa), and height (Kpa) were derived using the built-in parameters of the software. The mean colonic pressure formula was Mean (Kpa)–Min (Kpa). The number of peristaltic waves per minute was calculated manually. During the experiment, the temperature of the animal was maintained at 37 ± 0.5°C, using an electric heating board. The experimental procedure is shown in Figure 2.

### Transmission electron microscopy

The procedure is described as follows: Quickly fix the tissue with a volume of no more than 1 × 1 × 1 mm in electron microscopy fixation solution at 4°C for 2–4 h without mechanical damage such as traction, contusion, and extrusion and then rinse it three times with 0.1 M phosphate buffer (pH 7.4) for 15 min each. Fix it with 1% osmic acid (0.1 M phosphate buffer, pH 7.4) for 2 h at room temperature (20°C) and then rinse it three times as mentioned earlier. Dehydrate the tissue with gradient alcohol and acetone (50–70–80–90–95–100–100% alcohol-100% acetone-100% acetone for 15 min each time), and then dip it in agent and acetone 812 embedding (acetone: 812 embedding agents = 1:1 for 2–4 h, acetone: 812 embedding agents = 2:1 overnight, pure 812 embedding agents for 5–8 h) for penetration. Insert the sample into the embedding plate full of pure 812 embedding agents and place it in the oven at 37°C overnight and 60°C for 48 h. Slice the sample with an ultra-thin microtome (60–80 nm), stain it with 2% uranyl acetate saturated alcohol solution and lead citrate for 15 min each, and dry them overnight at room temperature. Transmission electron microscopy was used to observe and capture images. Image-pro Plus 6.0 (Media Cybernetics, Inc., Rockville, MD, USA) software was used for image analysis and data acquisition. One complete plexus was selected for each image, the number of unmyelinated nerves and plexus area (μm^2^) was counted, and the nerve density (/μm^2^) was calculated.

### Immunofluorescence staining

Frozen sections were used for immunofluorescence (IF) staining. Colon tissue was fixed in 4% paraformaldehyde overnight and dehydrated in 30% sucrose in 0.1 M PBS (Biosharp Life Sciences, China) at 4°C. After embedding in the optimal cutting temperature compound, the colon tissue was sliced into 10-μm thick sections and mounted on slides. The sections were then blocked in 0.2% Triton X-100 (Sigma-Aldrich (Shanghai) Trading Co., Ltd.) for 10 min and permeabilized in Sea BLOCK Blocking Buffer (Thermo Fisher Scientific, USA) for 1 h. They were then incubated with primary antibodies (nNOS, GB11145, 1:2000; ChAT, GB11070-1, 1:500) overnight at 4°C and incubated with secondary antibodies (nNOS, GB23303, 1:500; ChAT, GB25303, 1:400) for 1 h at 37°C. Finally, the tissue sections were covered by coverslips after washing them with 0.1 M PBS. Images were obtained by a fluorescence microscope (Olympus BX60 Darkfield DIC Metallurgical Microscope, Japan).

### Electroacupuncture intervention

The rats in the EA group received EA treatment on bilateral ST25 (Tianshu, located 5 mm lateral to the intersection between the upper two-third and the lower one-third in the line joining the xiphoid process and the upper border of the pubic symphysis) after gas anesthesia with isoflurane (2–5%; 9020000522; Shenzhen Ruiwode Lift Technology). Meanwhile, the same anesthesia was administered to rats in the PD group but without performing EA. For the EA group, two stainless steel acupuncture needles (20162270970; Suzhou HUATUO Medical Instruments, Suzhou, Jiangsu, China) of 0.2 mm in diameter were inserted at a depth of 5 mm into the ST25 acupoint. EA at ST25 was conducted with the HANS-100A (HAN ACUTENS WQ1002F; Beijing Anlong Photoelectric Technology, Haidian, Beijing, China) apparatus set to a waveform of the dilatational wave, a current of 2 mA and a frequency of 2/15 Hz, 20 min a day for 5 days a week, 1 week a course, and four continuous courses of treatment.

### Data analysis

**Figure 3:**
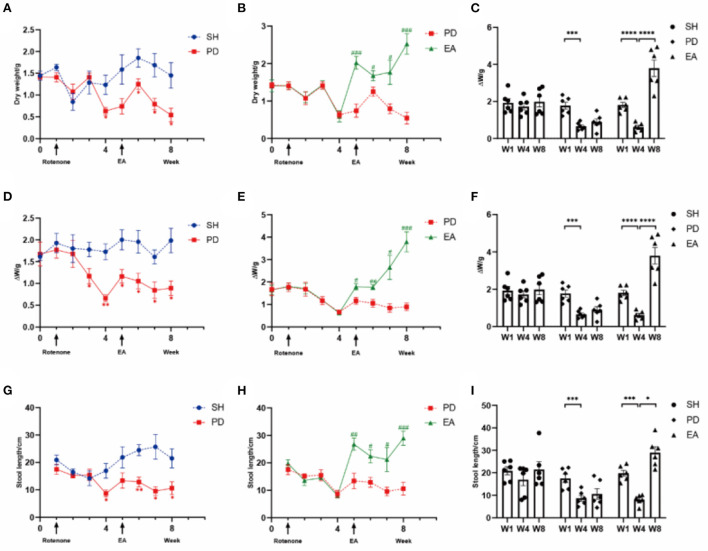
Excretion index of rotenone rats in different groups. Levels of **(A)** dry weight (Time × Rotenone Factor, *F* = 3.301, *P* < 0.01), **(D)** ΔW (*F* = 2.405, *P* < 0.05), and **(G)** length (*F* = 2.905, *P* = 0.01) in the SH group and PD group over 8 weeks (*n* = 6, *p < 0.05, **p < 0.01). Compared with the PD group, EA treatment increased **(B)** dry weight (Time × Rotenone Factor, *F* = 9.749, *P* < 0.0001), **(E)** ΔW (*F* = 10.02, *P* < 0.0001), and **(H)** length (*F* = 6.068, *P* < 0.0001) significantly (*n* = 6, ^#^*P* < 0.05, ^##^*P* < 0.01, ^###^*P* < 0.001). Comparison of dry weight **(C)**, ΔW **(F)**, and length **(I)** before and after rotenone or EA treatment in each group (*n* = 6, *p < 0.05, ***p < 0.001, and ****p < 0.0001). W, week; SH, the control group; PD, the model group; EA, the treatment group.

Data from all the experiments are expressed as mean ± standard error values. Weekly comparison of defecation indicators among groups in Figure 3 using two-way ANOVA. Paired *t*-test was used for comparison before and after rotenone treatment or EA intervention, and an independent *t*-test was used for comparing two different groups. All data analyses were performed using SPSS 23.0 software (IBM Corp., Armonk, NY, USA), and GraphPad Prism 9.4 (GraphPad Inc., La Holla, CA, USA) was used for data analysis. *p* < 0.05 was considered to indicate statistical significance.

## Results

### Effect of EA on representative symptoms of PD constipation replicated and whole gut transit time in rotenone rat models

**Figure 4:**
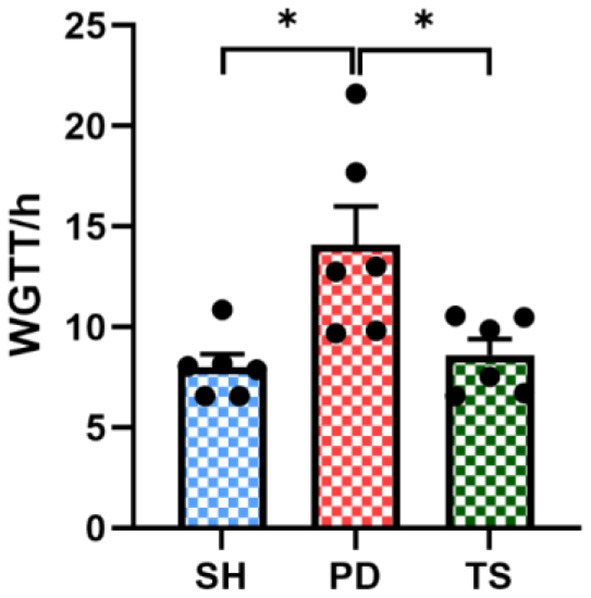
Whole gut transit time (WGTT) of rats after solvent, rotenone, and EA treatment (*n* = 6, *p < 0.05).

We measured stool dry weight, ΔW, and length to evaluate the successful induction of models. Compared with the SH group, rotenone injection significantly decreased stool dry weight (Figures 3A, C), ΔW (Figures 3D, F), and length (Figures 3G, I). Significant extensions of WGTT (Figure 4) were observed in the PD group, and the stool characteristics became dry, with smaller particles and brown-yellow color, which were consistent with clinical constipation. EA treatment increased stool dry weight (Figures 3B, C), ΔW (Figures 3E, F), length (Figures 3H, I), shortened WGTT (Figure 4), and recovered stool characteristics significantly compared with the PD group. Taken together, stool characteristics, output, and transit time were corrected in the EA group, suggesting the therapeutic effect of EA in PD constipation.

### Effect of EA on colonic motility of rotenone-induced rats

**Figure 5:**
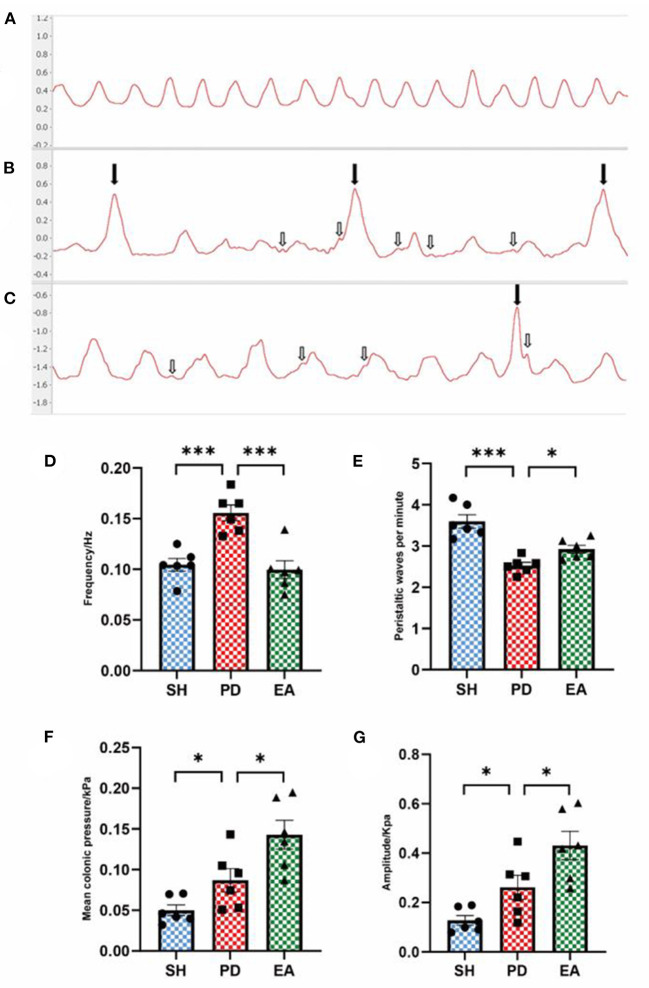
Effect of EA on colonic motility. Representative record of colon motion waveform in each group **(A–C)**. The frequency **(D)**, mean pressure **(F)**, and amplitude **(G)** of the PD group increased significantly compared with the SH group, and the peristaltic waves per minute **(E)** decreased. Compared with the PD group, EA treatment decreased the frequency **(C)** and increased the peristaltic waves per minute **(E)**. In contrast, the mean pressure **(F)** and amplitude **(G)** continued to rise (*n* = 6, *P < 0.05 and ***p < 0.001).

After EA treatment, the balloon method and the multi-conductive physiological recording system were used to record the colonic movement, and the mean pressure and movement frequency [the meaning of “frequency” is the overall frequency of colon activity, which is mainly influenced by meaningless irregular fibrillation (shown by the white arrow), rather than the density of peristaltic waves that represent effective movement.] of the colonic movement were calculated. The peristaltic wave in the SH group was continuous with uniform amplitude (Figure 5A). The PD group had irregular towering malformed waves (shown by the black arrow), and the interval between the two waves increased significantly (Figure 5B), suggesting that the rats in the PD group had intestinal peristalsis rhythm disruption and useless contraction. In the EA group, the continuity of the interval was restored and the abnormal waves were reduced but not completely disappeared (Figure 5C). Compared with the PD group, the colon motility frequency decreased and peristaltic waves per minute increased significantly in the EA group (Figures 5D, E). At the same time, we found that rotenone treatment increased the mean colonic pressure and amplitude of the PD group, while EA treatment did not reverse this change but further increased it (Figures 5F, G). The cause of this phenomenon will be analyzed in the discussion section in combination with the influence of the interference wave shown by the white arrow and the malformation wave shown by the black arrow on colon motion.

**Figure 6:**
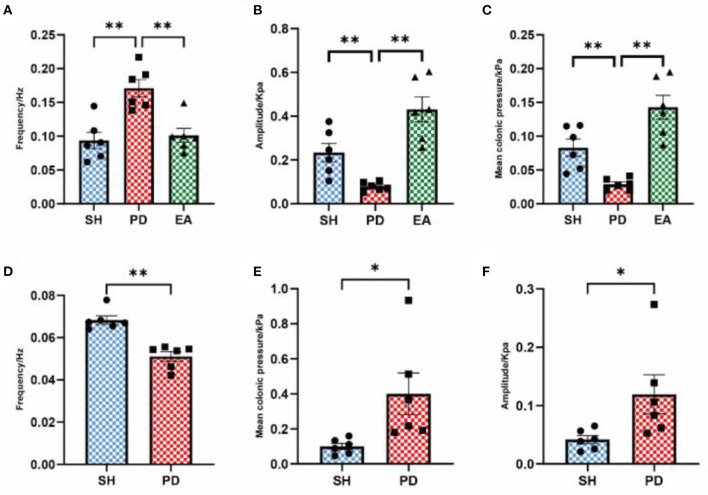
Effect of EA on colonic motility after parameter adjustment. The frequency **(A)**, amplitude **(B)**, and mean pressure **(C)** after eliminating the influence of abnormal waves. The frequency **(D)**, amplitude **(E)**, and mean pressure **(F)** after eliminating the influence of the vibration wave (*n* = 6, ^*^p < 0.05, ^**^p < 0.01). The actual meaning of the waveform without vibration wave is the peristaltic wave, which has been analyzed in the form of the peristaltic wave number per minute above (Figure 4E). Therefore, the comparison between the model and EA groups is not carried out here.

After the manual rejection of broad distorted waves, the frequency, pressure, and amplitude are collected and analyzed once more. The results showed that the mean colonic pressure and normal peristaltic wave amplitude decreased in the PD group and recovered after the EA intervention (Figures 6B, C), while the frequency results remain unchanged compared with the pre-adjustment period (Figure 6A). After adjusting the cutoff frequency to 0.1 Hz to exclude the effect of chattering waves, the frequency of the PD group decreased compared with the SH group (Figure 6D), while the mean pressure and amplitude results did not change (Figures 6E, F).

### Effect of EA on unmyelinated nerve fiber density and histopathology of colonic myenteric nervous plexus in rotenone rats

**Figure 7:**
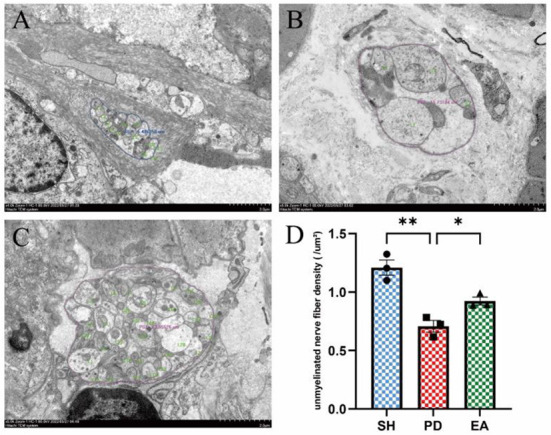
Effect of EA on colonic myenteric nervous plexus. Representative myenteric nervous plexus cross section under the transmission electron microscopy (Scale bar, 2 μm) of the control **(A)**, model **(B)**, and EA **(C)** group. Compared with the PD group, EA treatment increased the unmyelinated nerve fiber density **(D)** (*n* = 3, *P < 0.05, **P < 0.01).

We evaluated the effect of EA on the colonic myenteric nervous plexus by observing cross sections of the plexus under electron microscopy. Compared with the control group (Figure 7A), we observed a disturbed nerve structure in the PD group, with edema and sparse numbers of unmyelinated nerve fibers, as well as sparse and disorganized nerve microfilaments and microtubules (Figure 7B). After EA treatment, the nerve structure was improved. Compared with the PD group, the morphology of unmyelinated nerve fibers was more complete, edema was reduced, and the number of nerve filaments and microtubules was increased, while the nerve microfilaments and microtubules were neatly arranged (Figure 7C). We further counted the density of unmyelinated nerve fibers and found that the nerve fiber density in the PD group was lower than that in the SH group, while the nerve density increased significantly after EA treatment (Figure 7D).

### Effects on excitatory and inhibitory neurons of colonic myenteric nervous plexus

**Figure 8:**
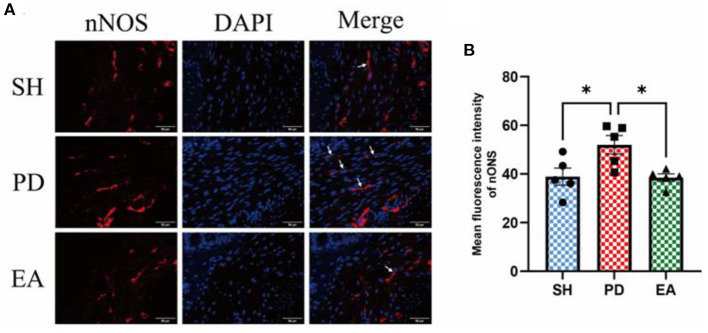
**(A)** Representative IF images of the colon. DAPI stained the nuclei (blue), while the red immunofluorescence represents the nNOS. The colon was observed under a microscope (×400 magnification). Scale bar = 50 μm. The three groups share scale bars. **(B)** Mean fluorescence intensity of nNOS. (*n* = 5, ^*^p < 0.05).

The results of IF in the colonic myenteric nervous plexus showed that nNOS expression in the colonic myenteric nervous plexus was significantly higher in the PD group compared with the SH group, while EA treatment significantly reduced nNOS expression in the myenteric nervous plexus (Figure 8).

**Figure 9:**
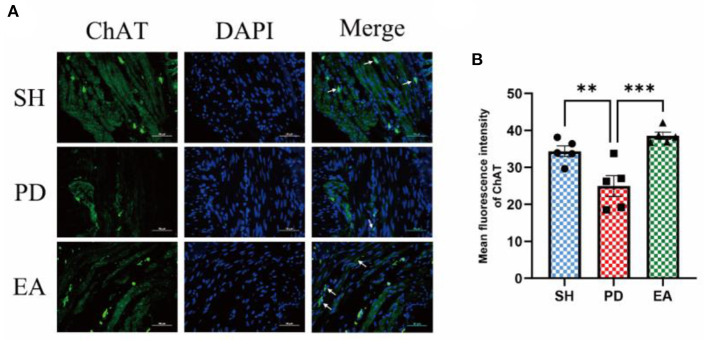
**(A)** Representative IF images of the colon. DAPI stained the nuclei (blue), while the green immunofluorescence represents the ChAT. The colon was observed under a microscope (×400 magnification). Scale bar = 50 μm. The three groups share scale bars. **(B)** Mean fluorescence intensity of ChAT. (*n* = 5, ^**^p < 0.01, ^***^p < 0.001).

The results of IF in the colonic myenteric nervous plexus showed that ChAT expression in the colonic myenteric nervous plexus was significantly reduced in the PD group compared with the SH group, whereas EA treatment significantly increased ChAT expression in the myenteric nervous plexus (Figure 9).

### Effects on the α-syn aggregation of colonic myenteric nervous plexus

**Figure 10:**
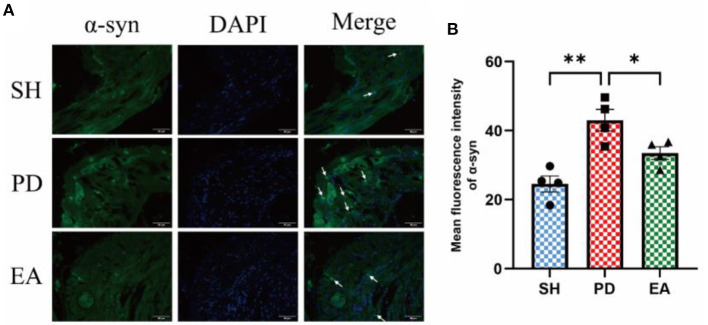
**(A)** Representative IF images of the colon. DAPI stained the nuclei (blue), while the green immunofluorescence represents the α-syn. The colon was observed under a microscope (×400 magnification). Scale bar = 50 μm. The three groups share scale bars. **(B)** Mean fluorescence intensity of α-syn. (*n* = 5, ^*^P < 0.05, ^**^P < 0.01).

The results of IF in the colonic myenteric nervous plexus showed that α-syn aggregation in the colonic myenteric nervous plexus was significantly higher in the PD group compared with the SH group, while EA treatment significantly decreased it (Figure 10).

## Discussion

In the present study, we demonstrated that rotenone disrupts ENS structure and function, resulting in prolonged WGTT and decreased mean colonic pressure and normal peristaltic wave density and amplitude in PD rats. Acupuncture treatment ameliorates ENS abnormalities and colonic dysfunction in PD rats, possibly by protecting the structural integrity of the colonic myenteric nervous plexus and balancing neurotransmitter expression.

In addition to typical motor dysfunction, NMS of PD is presented in more than 90% of people with PD (39). One of the key features of the prodromal phase, particularly constipation, is gastrointestinal dysfunction. Constipation is one of the most common and earliest forms of NMS, with a prevalence of up to 80% in people with PD. A total of 5% of patients develop constipation 10–20 years before the appearance of exercise symptoms. In addition, constipation may be a risk factor for PD, as men or women with constipation are two to five times more likely to be diagnosed with PD in the future (40). Therefore, constipation may be potentially useful for early diagnosis and therapeutic intervention in PD (41). The histology of PD is characterized by the formation of Lewy vesicles and Lewy neurosynapses, consisting mainly of intracellular aggregates of misfolded alpha-synuclein. Louis' vesicles are found in different areas of the brain and areas outside the CNS, such as the ENS (4, 5), and alpha-synuclein lesions can spread between the interconnected areas of the PNS and CNS, that is, the brain–gut axis. In Braak's classic hypothesis of the stages of PD pathogenesis, Lewy body lesions in the submucosal and myenteric nervous plexus neurons are at stage 0 of the disease process, that is, ENS lesions precede cerebral neurodegeneration. This suggests that ENS lesions play a very important role in the pathogenesis of PD. The ENS is the intrinsic nervous system of the gastrointestinal tract and consists of neuronal cell bodies or ganglia arranged in two nerve plexuses. The submucosal plexus is located between the mucosa and the cricothyroid muscle and mainly regulates secretion. The myenteric nervous plexus is located between the cricoid and longitudinal musculature and mainly controls smooth muscle movement. Each nerve plexus contains a complex, heterogeneous population of neurons. Neurons express and often co-express a variety of transmitters and neuropeptides, including acetylcholine, nitric oxide, vasoactive intestinal peptide (VIP), and dopamine (7, 42). The regulation of gastrointestinal function by ENS is due to the release of specific neurotransmitters synthesized by functional neurons. The main inhibitory neurotransmitters involved in the regulation of gastrointestinal motility are nitric oxide and vasoactive intestinal peptide (VIP), while acetylcholine (ACh) is the most represented excitatory neurotransmitter in the entire gastrointestinal tract. The control of bowel function by the ENS is based on the interaction between different neuronal subtypes in the ENS and the balance between the type of input and the level of postsynaptic receptor expression released into the neuronal network by each neurotransmitter (43). ENS degeneration is an important cause of gastrointestinal dysfunction during the prodromal period of PD. Therefore, PD and constipation can be treated with strategies to improve gastrointestinal function.

The efficacy of acupuncture in treating motor (12–14) and NMS, such as cognitive impairment (44), constipation (18, 19), insomnia (17), pain (45), and anxiety (15) in patients with PD has been widely supported by studies. Alternative therapies are reported to be used by 40% of people with PD, with acupuncture being the third most commonly used alternative therapy for PD (46). Acupuncture has a positive effect on relieving constipation symptoms in PD, although evidence from high-quality clinical studies is not yet available (18, 19). In addition to PD constipation, acupuncture is also considered an effective or safe treatment for functional constipation and other gastrointestinal disorders (25, 26). In terms of mechanistic studies, the absence of dopaminergic neurons in the nigrostriatal pathway is the most common pathological factor in PD. Acupuncture can mitigate brain dopaminergic neuronal damage through various pathways, such as apoptotic pathway, autophagic pathway, oxidative stress-related pathway, survival pathway, and neurotransmitter and its receptor and neurotrophic factor expression, thereby achieving neuroprotective mechanisms (15, 28, 29). In recent years, the gut–brain axis has offered a potential entry point for the treatment of PD as the gut–brain axis theory proposes a close connection between the gastrointestinal tract and the central nervous system (47). A growing number of studies have explored the feasibility of acupuncture in the treatment of PD by modulating gastrointestinal function (48). Ma et al. found that EA could improve PD-mediated neuropathy by promoting intestinal barrier repair and reducing intestinal α-syn deposits to inhibit neuroinflammation (49). Jang et al. showed that the enhanced motor function and protective effect of acupuncture on dopaminergic neurons in PD mice may be related to the regulation of gut microbial dysbiosis and thus the suppression of neuroinflammation (50). Another study reported that acupuncture may be beneficial in irritable bowel syndrome by modulating motor, visceral sensory, and/or gut–brain interactions (51). Considering the early damage to the ENS and the central substantia nigra striatal degeneration associated with dopaminergic innervation caused, there are no reports of acupuncture related to ENS lesions. In this context, we aimed to assess the effects of ENS structure and function on colonic motor patterns and associated neurotransmitter control in PD model rats and to explore potential mechanisms of action of acupuncture.

In this study, a model of rotenone induction was recruited to characterize colonic dysfunction and ENS injury. Compared with the MPTP (52) model, rotenone can reproduce the central and gastrointestinal features of PD in rats (53, 54), especially at low doses (2.5 mg/kg) of rotenone treatment, which has been further shown in mice to provide SNpc and myenteric nervous plexus replication of neurodegeneration and the presence of α-syn aggregates (55). Rotenone reliably induces PD models and has an effect on the gastrointestinal tract, which plays a role in the development of PD constipation models. In the present study, abnormal stool characteristics, excretion and water volume (Figure 3), and prolonged whole bowel transit time (WGTT) were observed in rotenone-induced rats (Figure 4), which is consistent with the clinical features of constipation and suggests the successful establishment of the model.

A previous study found *in vitro* that rotenone-treated rats exhibited physiological deficits in inhibitory neurons in the ENS, as evidenced by increased isometric contractility and reduced relaxation of the colonic longitudinal muscles (53). We have also discovered evidence in *in vivo* experiments that is consistent with this. We observed an increase in colonic frequency, mean pressure, and amplitude from the PD group (Figures 5D, F, G), and frequent towering distortion waves (Figure 5B) can be seen (shown by black arrows). Their amplitude exceeds that of normal creeping waves by a factor of more than one, making them easily observable. Another distinctive feature is the marked increase in the interval between two adjacent creeping waves (for convenience, we have named it the compensatory interval after the ECG waveform) and the decrease in the amplitude of normal creeping waves (see below for a detailed analysis), which means that the frequency (Figure 5E) and quality (Figure 6C) of valid creeping waves appear less frequently in the same period of time. The actual significance of this elevated distortion wave is not well-understood, as it appears to be a short period of intense bowel contraction. After we had removed this aberrant wave, the data were again collected and analyzed. The results showed that the mean colonic pressure and normal peristaltic wave amplitude in the PD group were reduced and recovered after the EA intervention (Figures 6B, C), while the frequency results were unchanged (Figure 6A). This is another confirmation that the efficiency of bowel movements is reduced in the PD group and is associated with the appearance of aberrant waves.

At the same time, we observed frequent irregular small fibrillation waves (shown by white arrows) in the main wave (creeping wave) and the compensatory interval. The amplitude of these small waves is not sufficiently different from the main wave to increase bowel movements. However, they are much larger than the main waves and are responsible for a large part of the increase in frequency in the PD group. As we observed a decrease in the number of creeping waves per minute in the PD group (Figure 5E), we further adjusted the parameters by adjusting the cutoff frequency to 0.1 Hz to exclude its effect. This suggests that this fluttering wave has a significant effect on the overall frequency of movement of the colon. It increases the energy expenditure of the colonic tissue but does not contribute to the propulsion of the intestinal contents. We hypothesize that it also had an effect on the decrease in mean colonic pressure and normal peristaltic wave amplitude in the PD group (Figures 6B, C). In summary, combining the results of constipation symptoms and whole bowel transit time, we hypothesize that although colonic movements in the PD group were hyperactive, they were mostly ineffective in nature. This is a reflection of a disturbance in the rhythm of bowel movement. The effect of EA on mean colonic pressure and amplitude shows an abnormal increase instead of a decrease (Figures 5F, G), due to the abnormal occurrence of aberrant and fluttering waves, which cause an abnormally high value in the PD group. This represents a pathological state of hyperactive and ineffective contraction in the PD group (Figures 6B, C). Unlike the PD group, the increase in mean colonic pressure in the EA group was not due to ineffective contraction. The increase in the density and amplitude of the peristaltic waveform was the reason for the increase in mean pressure compared with the PD group. As the bowel rhythm is restored, the increase in the index of colonic motility has a positive effect on the improvement of constipation.

To further test this hypothesis, we set our sights on excitatory and inhibitory neurotransmitters in the colonic myenteric nervous plexus. ENS inhibitory neurotransmission is achieved using non-adrenergic non-cholinergic pathways. NO is the main inhibitory neurotransmitter in the ENS and nNOS is the rate control enzyme for its production, mediating smooth muscle relaxation in the gastrointestinal tract, which is important for intestinal motility. Ach is an important excitatory neurotransmitter in the gastrointestinal tract, and ChAT is the main marker of cholinergic structure in the gastrointestinal tract. The IF results showed an increase in the expression of the inhibitory neurotransmitter marker nNOS in the colonic myenteric nervous plexus (Figure 8) and a decrease in the expression of the excitatory neurotransmitter marker ChAT in the PD group (Figure 9). In contrast, EA reversed this alteration. One study found that rotenone treatment did not alter the number of myenteric nervous plexus neurons (53). We believe that the integrity of the physiological function of the ENS is not only reflected in the number of neurons. The signaling between neurons is also essential. At the same level of neuronal numbers, if intercellular connections are reduced, the ENS is still in a state of reduced function. Therefore, we observed the density of unmyelinated nerve fibers in the myenteric nervous plexus by electron microscopy. The results indicate that rotenone treatment reduced the density of unmyelinated nerve fibers in the PD group, while EA reversed this state (Figure 4). In addition, we found significant α-syn aggregation in the colonic myenteric nervous plexus in the PD group, and EA treatment reversed this state. Although previous studies and our experiments have found that α-syn aggregation is mainly present in the submucosal plexus, α-syn is closely associated with pathological alterations in the myenteric nervous plexus as a major factor contributing to neuronal degeneration in Parkinson's pathology.

## Conclusion

In summary, subcutaneous administration of rotenone reproduces the clinical signs of constipation, delayed colonic transit, and ENS abnormalities in PD rats. EA intervention significantly improved stool characteristics and accelerated colonic transit in PD rats. This accelerating effect may be achieved by protecting the structural integrity of the ENS myenteric nervous plexus and balancing ENS excitatory and inhibitory neurons. These findings suggest mechanisms of ENS in PD in gastrointestinal motility disorders and the therapeutic role of acupuncture in PD combined with constipation.

## Data availability statement

The raw data supporting the conclusions of this article will be made available by the authors, without undue reservation.

## Ethics statement

The animal study was reviewed and approved by Scientific Investigation Board of the Nanjing University of Traditional Chinese Medicine, Nanjing, China (permission number. 202112A047).

## Author contributions

X-mH and BX: full access to all of the data in the study and responsibility for the integrity of the data and the accuracy of the data analysis. X-mH, BX, and NX: study concept and design. L-z-xS, XQ, and J-wD: acquisition, analysis, or interpretation of data. L-z-xS and YL: drafting of the manuscript. YL: critical revision of the manuscript for important intellectual content. L-z-xS and J-wD: statistical analysis. X-mH, BX, and ZY: funding, administrative, technical, or material support, and study supervision. All authors contributed to the article and approved the submitted version.
